# Basic emotions and adaptation. A computational and evolutionary model

**DOI:** 10.1371/journal.pone.0187463

**Published:** 2017-11-06

**Authors:** Daniela Pacella, Michela Ponticorvo, Onofrio Gigliotta, Orazio Miglino

**Affiliations:** 1 Centre for Robotics and Neural Systems (CRNS), School of Computing, Electronics and Mathematics, Plymouth University, Plymouth, United Kingdom; 2 Natural and Artificial Cognition (NAC) Laboratory, Dipartimento Studi Umanistici, Università degli Studi di Napoli Federico II, Naples, Italy; 3 Institute of Cognitive Sciences and Technologies (ISTC), National Research Council of Italy, Rome, Italy; Tilburg University, NETHERLANDS

## Abstract

The core principles of the evolutionary theories of emotions declare that affective states represent crucial drives for action selection in the environment and regulated the behavior and adaptation of natural agents in ancestrally recurrent situations. While many different studies used autonomous artificial agents to simulate emotional responses and the way these patterns can affect decision-making, few are the approaches that tried to analyze the evolutionary emergence of affective behaviors directly from the specific adaptive problems posed by the ancestral environment. A model of the evolution of affective behaviors is presented using simulated artificial agents equipped with neural networks and physically inspired on the architecture of the iCub humanoid robot. We use genetic algorithms to train populations of virtual robots across generations, and investigate the spontaneous emergence of basic emotional behaviors in different experimental conditions. In particular, we focus on studying the emotion of fear, therefore the environment explored by the artificial agents can contain stimuli that are safe or dangerous to pick. The simulated task is based on classical conditioning and the agents are asked to learn a strategy to recognize whether the environment is safe or represents a threat to their lives and select the correct action to perform in absence of any visual cues. The simulated agents have special input units in their neural structure whose activation keep track of their actual “sensations” based on the outcome of past behavior. We train five different neural network architectures and then test the best ranked individuals comparing their performances and analyzing the unit activations in each individual’s life cycle. We show that the agents, regardless of the presence of recurrent connections, spontaneously evolved the ability to cope with potentially dangerous environment by collecting information about the environment and then switching their behavior to a genetically selected pattern in order to maximize the possible reward. We also prove the determinant presence of an internal time perception unit for the robots to achieve the highest performance and survivability across all conditions.

## Introduction

The neuroscientific interest in understanding the basis of emotional behavior has been rising in recent years, in particular since the fall of the classic “limbic system” theory, which asserted that limbic areas were uniquely involved in the mediation of emotions and separated from the neural structures dedicated to cognition [1]. However, in the last two decades, the effort of studying the neural components of emotion has spread to research areas which are not strictly related to biology, such as robotics and AI, and the artificial systems developed to reproduce these circuits, along with the use of synthetic autonomous agents, have clearly showed to be particularly suited for simulating emotions [2]. When we speak of emotions from a purely computational perspective, as Canamero explains, we refer to all the features showed by the agents which constitute a behavior that can be labeled as emotional in comparison to the natural observed ones. But the concept of emotion is itself ambiguous in the psychological framework, since its definition floats in literature between a simple, observable physical arousal and a fully conscious experience of the affective state [3,4]. Regarding artificial agents, the lack of a recognized methodology brings up the debate about how much the emotional features should or should not be included directly in the design of these systems, or, in other words, what is the level of engineering and that of spontaneous emergence when we measure their emotional response [5].

Another issue which arises in the field of simulated agents is the lack of an agreed and reliable experimental paradigm for both a computational and a strictly neuroscientific investigation of the emergence of emotional behavior under an evolutionary perspective. Evolutionary theories, for example, consider cognitive mechanisms in terms of their adaptive functionality in ancestrally recurrent situations [6]. On the other hand, when we deal with artificial agents, we must consider that there are two different approaches to the definition of adaptation, whereas one defines the long term association between the environment and the agent, and the other concerns only how the agent approaches sudden changes, which reflects in a temporary and short-term adaptation [7,8]. While the former measures the emergence of an improvement in the result of actions over generations, the latter aims at simulating different decision making mechanisms in the organism’s life span.

Scheutz proposed a list of twelve possible roles of the emotion on the behavior of artificial agents, among which he lists action selection, adaptation, sensory integration, motivation, goal management and learning [9] and several have been the attempts to engineer the above strategies in autonomous agents, in particular to investigate the relationship between emotions, behavior and decision-making. Gadanho and Hallam reported the outcome of an agent taught with reinforcement learning and embedded with four basic emotion (fear, anger, happiness and sadness) and an essential “hormone system” based on the somatic marker hypothesis [10]. The emotions’ activations, represented by units in a recurrent network, were determined by input units labeled as feelings, such as, among others, hunger, pain and proximity, directly measured from the agents’ sensory perception. These bodily perceptions inputs in the network also determined the behavior of the “hormone layer”, which then affected the emotion layer connections, modulating the dominant emotion output. The agent showed appropriate transitions between affective states according to the situation [11]. While the results of this early study, that reported an accurate specialization and balancing of the emotion units in the environment, supported the idea that a simplified emotional system can potentially improve the ability to adapt and the performance of artificial agents, it is important to stress that the possible affective states that the agents could express were already defined as separate nodes within the network and lead to a specific action, therefore not allowing the spontaneous emergence of an adaptive behavior *per se*. This and other subsequent studies tended to embed the emotions using a similar model, i.e. by setting a certain emotion as a layer or node that leads to a stereotyped behavior and the analysis is conducted only on the ability of the network to learn its proper activation. Tsankova, for example, recently investigated the impact of simulated emotional and immune system on the behavior of artificial agents equipped with neural architecture inspired on the amygdala structure in a goal-following task [12]. Another recent study by Jha and colleagues described the design of a situated robot with basic decision-making abilities and tested its behavior in an artificial environment. The robot was asked to select one among different tasks in order to increase its Comfort level. The simulations were conducted using the multiagent emotion generation engine proposed by Godfrey [13,14].

While the mentioned–and other–studies have successfully showed the primary role of emotions on the organisms’ survivability and adaptation when introduced in artificial systems [15,16], poor has been investigated on the concept of emotion as an emergent factor from the interaction between the agent and the environment. Most of the authors’ focus has concentrated on measuring the agents’ ability to choose one behavior over another in several specific contexts, leaving out the possibility to observe the appearance of new unsupervised behavioral strategies used to cope with unpredictable—but recurrent—problems. This casual appearance is exactly how evolution, thanks to random mutations, shaped simple and complex adaptive mechanisms in natural organisms throughout generations. So, if we wish to dig more into this genetic aspect, and simulate how these adaptive strategies have evolved, we need to build a computational model which can be, in its simplest features, compared to the process of natural selection, and this can be achieved with the use of genetic algorithms and by leaving the agents free to explore and adapt, rather than to choose a behavior over a predefined set. In this study we will present a bio-inspired computing methodology to investigate the emergence of emotion-driven behavior in agents equipped with artificial neural networks (ANNs) and trained with genetic algorithms. The setting we use for our simulations is a fear-based context discrimination task, which is also suitable for the reproduction of the well-known Pavlovian threat conditioning (PTC) [17]. The simulated populations of agents are evolved throughout generations to learn to “keep” or “discard” each stimulus perceived by their visual system in their environment. The percentage of stimuli to keep or to discard depends on the condition. In case the robot decides to go for the wrong action, an electric shock is administered which determines a loss in terms of life duration. The simulated environment contains elements typically belonging to classical fearful situations, and the model of the “emotional circuit” activation is based, in its minimal aspect, on the response to a stress experienced by a natural organism. The particular difference we wish to stress is that the stimuli are all equal to each other, therefore the robot must learn to discriminate the “dangerous” context from the “safe” one and to “cope” with the “sensations” perceived by its units. Robots can choose between keeping, discarding or ignoring the stimuli but also need to learn to balance their need of exploration, and the resulting new, unpredictable behavior relies on the internal sensation of danger and safety acquired throughout past experiences of the individual and of its ancestors. The interaction between the agents’ choice of action and its effect on the internal sensation units guides the evolution of the fearful behavior of robots and their offspring, leading to the emergence of specific strategies in order to recognize and avoid the danger.

In a preliminary study, we introduced the experimental paradigm in its basic features, analyzing the performance of four different fully-connected neural network architectures and testing two fitness functions for the genetic algorithm on this task. Results showed significant advantage of the processing speed in feedforward networks that led to better performance [18]. Here we propose an architecture that allows the robot to gather information from the environment and learn how to use these cues to discriminate different contexts in the presence of the same visual stimuli, triggering an appropriate fearful or approaching behavior. We aim to investigate the emergence of a self-organized phylogenetic system and evolve multiple populations equipped with different architectures, tuning the fitness function and the parameters for the training and the evolution.

### The role of emotions in nature

The definition of emotion is not unique in the psychological and neuroscientific literature. For this reason, it is necessary to clearly define the theoretical framework we referred to in order to operationalize the concept of "emotion" and "fear". The first categorization of emotions distinguishes basic emotions, as happiness, anger or disgust, from social emotions, that are more complex in that, emerge later in ontogeny [19] and depend on interpersonal relationships, like thoughtfulness, boredom, compassion, guilt or admiration [20–23]. Another distinction to be made is between the cognitive aspect of the emotion, i.e. the subjective experience of the situation, and the pattern of individual autonomic responses to stressful conditions, the latter not being necessarily conscious, but tied to a specific trigger situation. There is a debate about what actually constitutes this group of responses and the level of complexity of these reactions, in particular their modularity and their interpretation. Specifically, there are two different approaches to this problem. One defines the emotion as permeated necessarily by both psychological and physiological aspects, whose combination allows the identification of emotions as universally similar in their components in animals, humans or robots, with different underlying neural structures but achieving the same manifestation [3]. This view, recently brought forward by Adolphs, embraces also a dimensional perspective of emotions, that are categorized according to their position on theoretical continuums of diverse dimensions, each comprising two sub-categories: similarity (intensity and valence), flexibility (persistence and learning), coordination (hierarchical behavioral control and multi component effects) and automaticity (priority over behavioral control and poised for social communication) [24]. The second approach, which only partly contrasts with the former, considers emotions as arising from the co-occurrence of spatio-temporal events, in that the underlying brain structure and activation is necessary but not determinant to the identification of an emotion. In particular, the input perceived by the brain is interpreted according to its similarity with past sensorial experiences, a process called “predictive coding” [25]. This prediction, computed using previously constructed internal models, categorizes the sensations providing a meaning and allows the implementation of a specific action plan. Then, the error between the prediction and the actual input is propagated and used as feedback to update the internal model. This constructionism distances itself from the classical view that assumes that emotions have an innate “essence” that distinguishes them, but also from a dimensional model. Actually, the theory of constructed emotions is based on the idea of the brain as a concept generator, that collects representations; therefore, for any perception, affect or emotion, a cortical mediation is needed [4].

However, both views are based on the evidence that a single organ in the brain does not control the expression of only one emotion. Considering that in the brain there is not a one-to-one mapping between regions and behaviors, every emotion, considered as a process, consists of the emergence of a pattern from the interaction of multiple interconnected areas. These networks themselves can participate in several other behaviors [26], and the general view that encompasses this cerebral networks’ plasticity across physical and cognitive processes is called “neural reuse” [27]. What determinates the outcome during a specific event will depend on situational variables, involving, for example, the organism's arousal or the temporal pattern of the danger. Cacioppo and Tassinary [28], among others, proposed a mapping between psychological processes and physiological ones taking into account a spatiotemporal pattern of the co-occurrence of physiological indices. The involvement of the amygdala in several affective processes has been also extensively proved [29–31].

Both views also agree on the concept that emotions emerged during evolution [32] and on the concept of degeneracy, that defines each emotion as created by multiple spatiotemporal patterns in populations of neurons that can vary among individuals and time and type of the specific trigger event [4,24]. It is indeed the network structure of the brain, from a molecular and molar point of view, that allows the emotional response, but also its temporal occurrence and the surrounding environment. In this paper, we embrace the definition of emotion state shared by Adolphs, LeDoux and other researchers, which consider emotions as adaptive events or states that are likely to occur in humans and animals, but that may or may not have a subjective component, depending on the species and situation involved [33,34], and that therefore do not require awareness nor a cognitive experience. In the case of amygdala, for example, recent neuroscientific evidences support this hypothesis [35], and recently a distinction between attentional unawareness and sensory unawareness was proposed [36,37]. Even though most studies in the field of affective neuroscience rely on brain imaging techniques that do not track the modifications of the brain activation in a specific time period (e.g. fMRI or PET), other important evidences can be obtained when considering the temporal aspect of the emotional experience (e.g. measured using EEG). Anger and fear, in particular, can impact survivability only if they occur promptly [36]. A recent study by Costa and colleagues supports the idea that fear is processed earlier than other basic emotions [38].

### Evolutionary view of emotions

In contrast to the dimensional theories of emotions, which declare that affective states are produced by a combination of the same elementary components [39], the most agreed being the type of valence and amount of arousal [40], basic discrete emotion theorists believe that there exists a set of innate affective “programs” shared among different organisms that appear to be similar and comparable across different cultures and societies [21]. These emotions, according to the evolutionary theories, were selected through evolution in order to promote the survivability of the species in their specific primitive environment. In the case of fear, an example may be the situation of being alone at night, that triggers the activation of a detection circuit that tries to perceive possible cues of the presence of a threat [41]. Each ancestrally recurrent situation activates an associated “emotional program” which in the past led to the best adaptation to the cluster of repeated events and conditions posed by the environment. In order to distinguish the emotions which can be considered as elicited by a recurrent situation in the past from higher cognitive emotions, Panksepp proposes the distinction between primary, secondary and tertiary processes, whereas the first type, which he recognizes as being rage, seeking, lust, fear, care, panic/grief and play, have been found in rats and do not necessarily involve cognition in order to arise [42,43].

The main feature by which a specific strategy is selected throughout generations is its function, and for functional analysis we intend the investigation of how an individual’s present behavior leads to the maximum fitness [44,45]. This functionalism extends the possibility that other states such as coma, shock, or confusion could have been evolved as modes of operation triggered when an organism is coping with illness [41]. Regarding the emotion of fear, however, there is in general more agreement on its evolutionary basis, since it can also be proved by the fact that mammalians exhibit basic fear responses even before having experienced pain or danger themselves [46].

### The concept of fear

The reason why fear is by far the most studied emotion in humans and animals depends on several factors, among which the easiness of eliciting aversive affective states in rats, the similarity of the response pattern of fear among mammalians and the agreement reached upon the role of the amygdala in processing aversive stimuli and conditioned fear responses. Lately, much effort has been made in the field of neuroscience in order to clearly define the circuits involved in the genesis of affective states in animals. While the boundaries of the "limbic system"–if the concept is not yet abandoned—have been trespassed turning it into a fading region which includes some parts of the mid-brain and neocortex [47], there is good evidence about the role of the amygdala in the emotion of fear [48–50]. The principal paradigm for the investigation of fear is Pavlovian threat (or fear) conditioning [17]. In PTC, the subject receives a neutral conditioned stimulus (CS), followed by a stressful unconditioned stimulus (US). In the case of an auditory input, for example, a CS can be a tone, and the US a footshock. The stimulus is, in the first instance, perceived by the sense receptors and sent to the sensory thalamus and then to the cortex. After the pair of CS and US has been presented a few times, the CS elicits the same physiological response which occurs in the presence of the US [51]. The pairing of the two stimuli, in fact, appears in the lateral nucleus of the amygdala (LA) thanks to the incoming signals from the cortex and the thalamus. The LA, specifically in its dorsal region, is connected directly with the central nucleus (CE), which then sends the information of exposure to the CS to the hypothalamic and brainstem areas which process the autonomic responses [1,52–54]. Basal and accessory basal nuclei of amygdala do not appear to be involved in CS conditioning [55]. This two-pathway connection received by the lateral nucleus of the amygdala is crucial for a rapid encoding and response to the conditioned and unconditioned stimulus, which can be elicited prior to cognitive processing. Among the species-typical responses to fear in rats–but that extends similarly to a great variety of mammalians—we cite freezing, variation of heart rate and blood pressure along with ADH and ACTH hormone secretion. Bechara and Damasio proved the centrality of the role of the amygdala nuclei also in humans [29]. Another consequence of fear conditioning is the association of unconditioned responses also to the environment where the CS-US pairing takes place. This process is termed “contextual fear conditioning” and basal nuclei of amygdala appear to be involved [56]. As previously mentioned, the activation of the amygdala is necessary but not sufficient to establish the observable affective response. In order to define the emergency of different processes from similarly interconnected areas, Pessoa proposes the difference between structural and functional connectivity, whereas structural responses in two regions could be correlated, but the effect of intermediate regions and the context in which the stimulus appears will determine the functional response [26]. Structural interconnectivity itself can only allow the prediction of few features of the emotional behaviors. For example, considering that anger responses share the negative valence and the high arousal typical of fear responses, the distinction between the two is operated on the basis of the behavior adopted by the organism, which in fearful situations consists of freezing and/or avoidance, while in case of anger is characterized by aggression and closeness.

Few have been the studies that tried to propose a computational model of the neural connections able to determine the emotional response. Among these, Morén and Balkenius described a simple neural network model which included all the most relevant neural structures that allow fear conditioning, i.e. amygdala, thalamus, orbitofrontal cortex and sensory cortex. Each of these was represented by a group of three or more interconnected neurons and had a specific function such as capturing external signals, evaluating the intensity, learning the association and allow/inhibit the emotional response [57]. Another model, recently developed by Navarro-Guerrero and colleagues, aimed at proposing a possible self-defense system for autonomous robots [58]. The architecture was composed of an echo state network, which represented their simulated prefrontal cortex, and its connections to a feedforward network, that constituted various units of the amygdala nuclei, the thalamus and the auditory system. This network was also tested in a physical robot (NAO). Even though the theoretical network models discussed above represent a valid simplification of the structures inside the brain involved in the genesis of emotions, what lacks to these approaches is an attempt to explain how each of these modules specialized to their current, assigned role in the genesis of the emotion. Since both the networks were already organized in layers, there was no space left to the emergence of a self-organization of the nodes into different structures and what is considered as “learning” can be exclusively seen as an ontogenetic change, leaving aside the possibility of observing an autonomous and evolving organization of the parts through time. The present study wishes instead to try to fill this gap, proposing a system which aims at modeling the emergence of fearful behavior in its evolutionary aspect, opening up to the future investigation of the spontaneous specialization of each node of the network. Other than that, our model helps investigate the temporal evolution of the activation during the emotional response, an aspect of neglected in neuroscientific studies which do not involve a measurement through time. We had previously started to explore the efficacy of genetic algorithms for the simulation of a phylogenetic evolution of the animal’s emotional system, taken in its simplest aspect, and we showed how a typical “fear” response, the avoiding behavior, is observed as a result of learning. We wish to use the same experimental paradigm to present a model of the evolution of the “fear” system as a result of learning danger from environmental cues.

## Materials and methods

In our preliminary study, we described the methodology used to build the framework in which the artificial agents are tested. In particular, we used simulated robotic agents equipped with a neural network and trained them with genetic algorithms to learn to recognize dangerous from non-dangerous conditions by picking up or avoiding stimuli of different colors. We evolved populations of robots for 1000 generations and compared the outcome of four architectures, a shallow feedforward network, a recurrent neural network, a recurrent neural network with the additional recurrence of the motor neurons and a network with the only recurrence of the motor neurons, and evaluated their performance using different fitness functions. We observed that, in the unsafe conditions, dangerous stimuli were able to trigger an avoidance behavior which resulted in both navigating around them and refraining from picking them up. We found out that the agents who could achieve a higher score and make less errors were those equipped with the feedforward network. [18]. Some details of the fitness function we used and of the genetic algorithm are maintained in this study and are described below.

**Fig 1:**
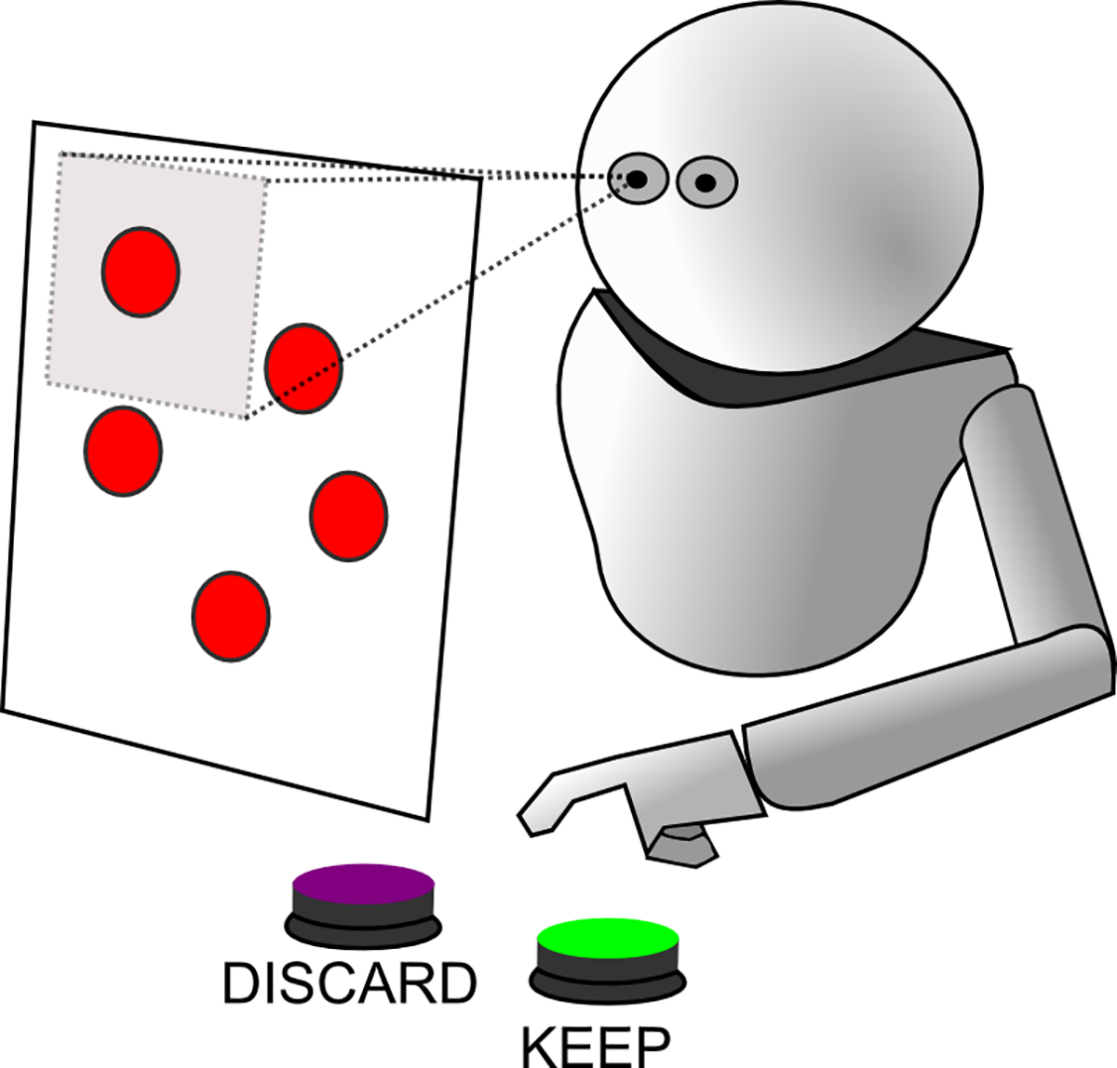
The iCub experimental setting. The simulated experimental setting, with the iCub robot moving its hand on the action buttons while exploring the stimuli on the squared board in front of it.

To simulate the artificial agents, we used a modified version of the Evorobot* platform, an artificial life simulator developed by Nolfi and Gigliotta [59] which allows the simulation of specific aspects of a range of different robots, like e-puck, Khepera robots and, in subsequent releases, the iCub robot [60]. This open-source software is composed of the following features: 1) a neural network library, which allows epoch training with genetic algorithm; 2) a robot simulator, which connects the neural structure with the specified sensors of the selected robot; 3) a tool for simulating an artificial environment with the possibility of adding collision units or stimuli; 4) a tool for transferring the evolved network into the real robot. The simulated robot we used is based on the iCub humanoid robot features, in particular for pointing abilities and visual exploration. The iCub is mainly used for experiments and simulations in the field of cognitive sciences, and its body resembles that of a 3-years old child [61]. For our experiment, we integrated in the simulation its visual system and finger-pointing abilities. The robot is controlled by a neural network and is equipped with a sensory-motor system composed by a pan-tilt camera. The visual system represents an evolution of the artificial retina described by Floreano and colleagues, and allows the agent to perceive a visual scene of 100x100px. Thanks to this sensory system the agent is also capable of discriminating the luminance of the stimuli on the scene [62]. The environment is composed of a 400x400 pixel whiteboard per each trial and the robots are free to visually explore it during their lifecycle. The board contains colored stimuli–represented by circles–randomly positioned on the screen. We used low luminance (dark red) stimuli for all the training conditions, except for one experiment, in which we included also high luminance (light red) ones. Depending on the trial, the same stimuli can be safe or dangerous for the robot’s health. The robot, with its hand, can reach to 2 different buttons that perform an action on the stimulus currently perceived by the retina and that are posed in front of him, one is for discarding and the other for storing. The decision about which action is performed is controlled by the neural units; if it presses the button to store a safe stimulus or discard a dangerous stimulus, it gains fitness; instead, if it stores a dangerous stimulus or discards a safe one, receives an electric shock which determines a loss in terms of life steps. Fig 1 shows a graphical example of the experimental setting. The image displays the robot moving its visual system on the squared board containing the stimuli and holding its hand near the two action buttons. This setting is inspired to the Pavlovian threat conditioning framework in that there is the analysis of how the agents adaptively learn the association between the perceived stimuli, the action and the outcome of the action in terms of fitness, which varies according to the condition and time.

**Fig 2:**
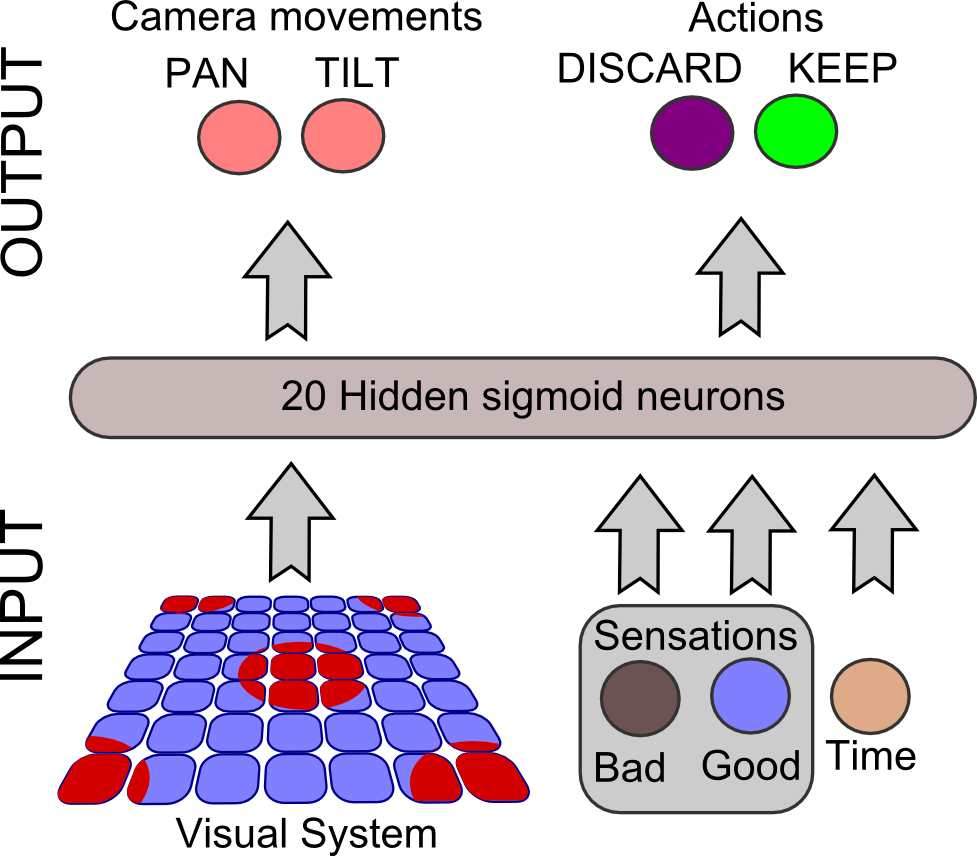
The neural architecture. The structure of the fully connected neural network used in the different conditions. The input layer, composed of 49 visual neurons, 2 sensation neurons and 1 clock neuron; the hidden layer, composed of 20 sigmoidal units; the output layer, composed of 2 motor neurons and 2 actuator neurons.

**Fig 3:**
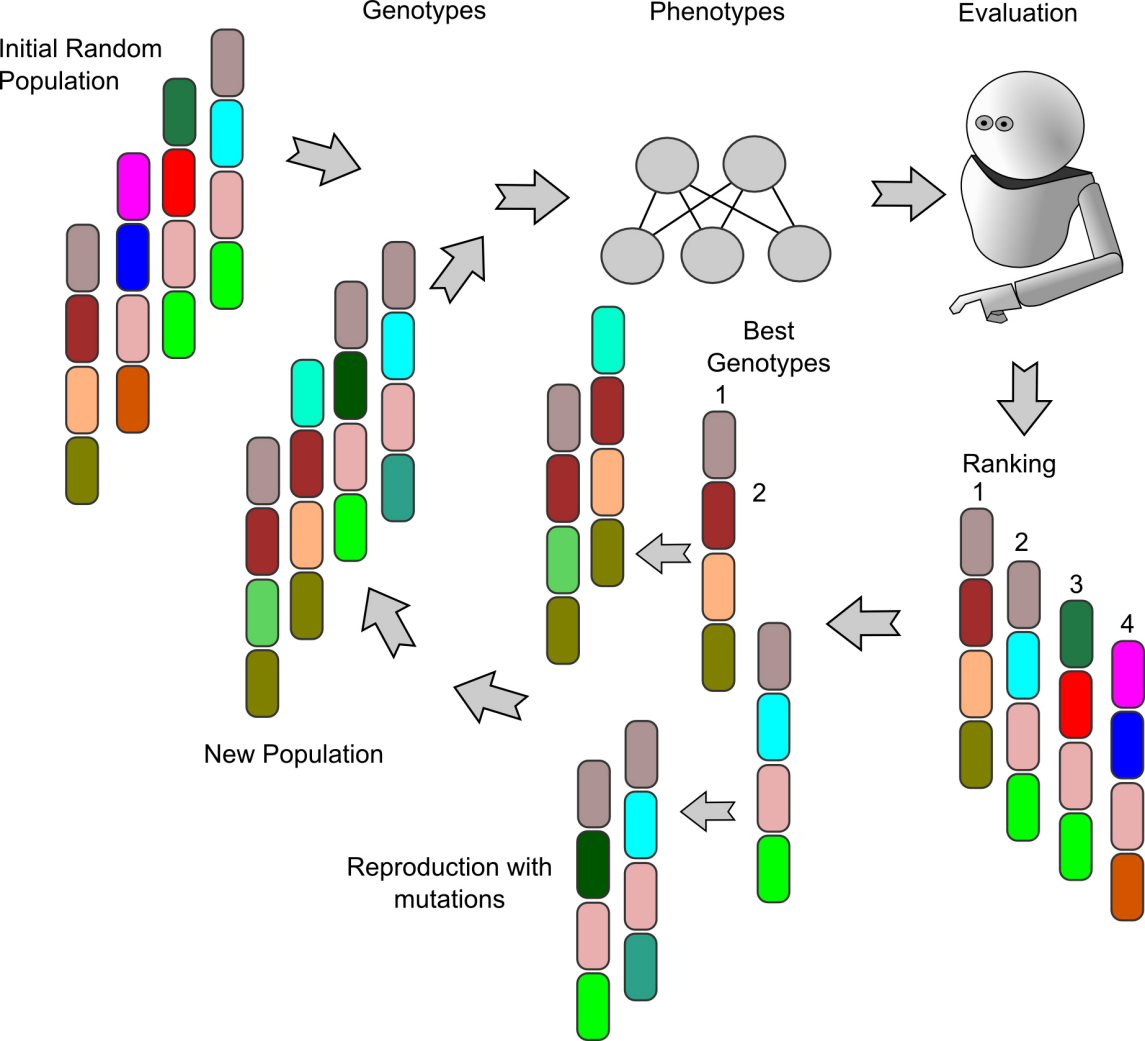
Description of the genetic algorithm. The figure summarizes all the steps of the genetic algorithm during a single generation: 1) an initial random population of 100 individuals composed of 8-bit genotypes is generated; 2) the genotypes of the initial population are expressed into phenotypes for each individual, i.e. the actual networks’ weights and biases; 3) the individuals’ phenotypes are evaluated during the training phase by measuring their behavior with the fitness function; 4) the fitness values obtained by each population’s individual are ranked; 5) the 20 best individuals are allowed to reproduce and to generate an offspring of 100 individuals by flipping the original genotypes bits with a 0,01% mutation rate; 6) the offspring constitutes the new generation which can go through the selection cycle again.

The neural network architecture across all the experiments is showed in Fig 2. In detail, it is equipped with the following separate input units: 1) 49 visual neurons organized in a 7x7 grid which constitute the squared area of the retina; 2) a “safe sensation” unit, which increases its value *S* if the correct button is pressed on the stimulus (discard if dangerous and store if safe) according to the following logistic function:

$$S(n)=\frac{1}{1+e^{-\beta \frac{n}{N}+\alpha }}$$ Eq 1

where *β* = 10, *α* = 3, *N* represents the total number of stimuli in the scene (in our case *N* = 16, which represents the same number of stimuli used in the framework established in our previous experiment) and *n* the number of correct actions actuated on the stimuli. We decided to normalize *n* on *N* so that the agent’s “safety” sensation grows proportionally to the amount of total stimuli and cannot be saturated before all the items are explored; 3) a “fear sensation” unit, which increases its value if the wrong action is selected and follows the same rule as **Eq 1** with *n* representing the number of wrong actions on the stimuli (discard if safe and store if dangerous); 4) a “time perception” or “clock” unit, which increases its activation value *C* according to the time passed since the beginning of the trial and follows the function below:

$$C(t)=\frac{1}{1+e^{-\beta \frac{t}{T}+\alpha }}$$ Eq 2

where *β* = 10, *α* = 4, *T* represents the total number of the robots’ time steps per trial (in our experiments *T* = 1000) and *t* the current time step, which is reset at the beginning of each trial. In our case, since both time and actions selected cannot hold negative values, we chose the *α* and *β* parameter so that S(0) ≈ 0, S(16) ≈ 1, C(0) ≈ 0 and S(1000) ≈ 1. The hidden layer consists of 20 sigmoidal units fully connected to the outputs, that are composed of: 1) 2 motor neurons which control the pan/tilt movements of the retina; 2) the actuator which triggers the hand movement of pressing the “store” button; 3) the actuator which triggers the hand movement of pressing the “discard” button. The idea of the sensation units is considered as a more complex form of the threshold “emotion” unit described by Parisi et colleagues, who attempted to design a neuron evolved and specialized to determine the cancellation of a stimulus overwriting the output value of other units in the network [63]. Regarding the genetic algorithm, a population of 100 random genotypes encoding weights and biases of the related neural network is created at the beginning of each evolutionary experiment. Genotypes, constituted by 8-bit genes, are then mapped onto neural controllers’ parameters in the range [–5,5]. Individuals equipped with genetically encoded neurocontrollers are then evaluated according to their ability to make the correct choice in different scenarios of the task. The first 20 individuals, ranked according to their fitness function, are used to repopulate a whole new generation of 100. Mutation is applied by flipping genotypes’ bits with a probability of 0.01%. The process iterates for each generation and is briefly summarized in Fig 3. All the parameters of the experiments are consistent with the findings of our previous study.

### An architecture comparison

For our study, 10 populations of each neural architecture and condition were evolved for 5000 generations. Each generation was trained on 30 trials consisting of 16 visual stimuli each; the trials were divided into triplets, for a total of 10 triplets. Each trial could belong to the “dangerous” or “safe” condition: the dangerous condition contained 100% of dangerous items and therefore the right action to learn was to discard all of them, while the safe condition contained 100% of safe items, and the robot had to learn to store them all. The generations were trained on two different trial sets: the first half– 2500 generations–was trained on 10 triplets of trials composed by 1/3 of dangerous trials and 2/3 of safe trials and the other half was trained on 10 triplets composed by 1/3 of safe trials and 2/3 of dangerous trials. The stimuli were all visually equal to each other by size and color except in one condition, where half the items were randomly displayed as darker than the others but this visual information was not related to the safety or danger of the stimulus itself.

During the trial, the robot was free to move its visual system for 1000 time steps, and the current time step was defined as *t*. If a target was perceived, three were the possible outcomes: if the activation *r* of either of the actuator units reached at least the value *r* = 0.7, the corresponding action (store or discard) was executed on the item, the robot had a loss of 1 time step (*t* = *t* +1) and the stimulus disappeared from the board; if both the actuator units had a value over *r* = 0.7, no action was performed on the item but the robot had a loss of 1 time step; if neither of the actuator units value reached the threshold, the stimulus was considered as ignored. In case the correct action was selected on the stimulus, the robot gained a reward *R* depending on time *t* according to the following fitness function:

$$R=T\left(\frac{1}{1+e^{-\beta \frac{t}{T}+\alpha }}\right)$$

where we set *β* = 10 and *α* = 4. If either of the actuator units reached at least the value *r* = 0.7 when the robot is not perceiving any stimulus, the robot had a loss of 1 time step for each of the active unit (maximum *t* = *t* +2). In case the trial ended without gaining any reward, the robot was punished with a loss *L* = *T*. The fitness was evaluated as the sum of the rewards gained by the individuals at the end of the 30 trials. The reward is set as dependent on *t* since we wish to allow an initial “neutral” exploration in which the agents are free to either keep or discard the items they encounter and experience the outcome of these actions on their sensation units, just like young animals test their innate adaptive or maladaptive strategies in a controlled environment before to take the real risks.

Five different neural architectures and conditions have been used in the training phase: 1) feedforward networks (ANN); recurrent networks (RNN); feedforward networks without the time input unit (ANNnoTime); recurrent networks without the time input unit (RNNnoTime); feedforward networks which were tested in the same conditions but which perceived dark luminance stimuli in half the trials and light luminance stimuli in the other half (ANNColorInvariance). While the first four conditions aimed at investigating the impact of the recurrent connections on the perception of time and performance on the task, the last condition aimed at testing if the network was able to generalize its ability to discriminate the dangerous and safe contexts in the presence of a disturbance variable like the luminance of the stimuli.

## Results

**Fig 4:**
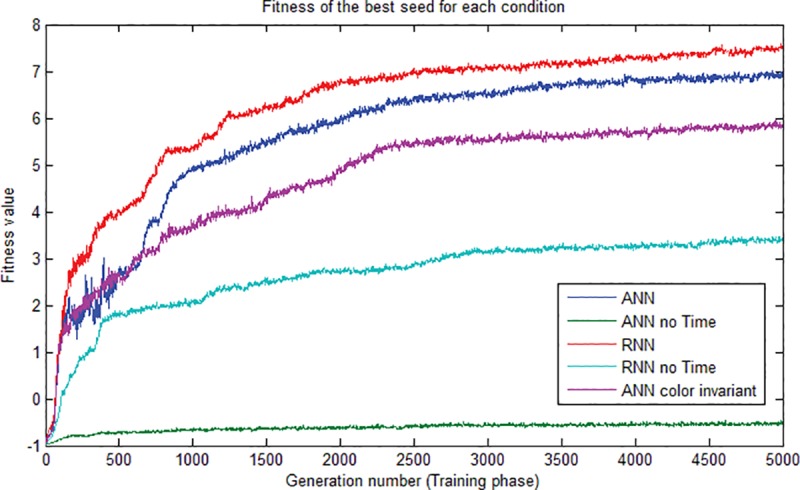
Comparison of the architectures’ fitness functions. The plot shows the difference in growth of the fitness function of the best individual for each of the conditions during the training phase. Apart from the ANNnoTime condition, the other architectures all scored a fitness above 2.0.

Before to test the different networks on the test set and have a look at the performances, the growth of the rewards during the evolution were analyzed. The plot of the fitness growth obtained by the best individual in the 5 different test conditions until the 5000^th^ generation is showed in Fig 4. The functions are all significantly different from each other (p-level < 0,05) as summarized in Table 1. LSD and Tukey post-hoc tests on the ANOVA were also conducted but are not reported here for brevity. First of all, the curve trend of the plots appears not to be affected by the change in the training set which occurs at the 2500^th^ generation. The highest score is obtained by the RNN equipped with the time input unit, which at the end of the training reached a fitness of 7,51. The others reached, from the best to the worst, 6,943 (ANN), 5,885 (ANNColorInvariant), 3,39 (RNNnoTime) and -0.501 (ANNnoTime). Apart from the reward trend, the main investigation needs to be conducted on the behavioral strategies and the differences of performances emerged among the various architectures. Since the agents could use no visual cue for deciding whether the condition was dangerous or safe, but had to rely exclusively on their sensation units, it becomes important to evaluate how these activation values shaped into behaviors which led to scoring such a high reward and guaranteed an adaptation for both the conditions. Furthermore, since the earliest steps do not provide a significant reward, it is relevant to explore the activation patterns throughout the time steps of the action units and try to understand the strategies behind the “store” or “discard” actions performed by the agents. For the test phase, we extracted the best individual for each of the five conditions and tested them on 1000 trials, of which 50% belonged to the safe condition and 50% belonged to the dangerous condition. The stimuli were 16 per trial and all had a dark luminance. Table 2 shows the performances of the architectures on the correct and incorrect actions in the safe condition (2.a and 2.b) and in the dangerous condition (2.c and 2.d). The architectures which achieved the lowest number of incorrect actions (Table 2.b and 2.d) and the highest number of correct actions (Table 2.a and 2.c) are highlighted in bold. Those who scored the highest number of incorrect actions and the lowest number of correct actions are highlighted in italic. The first thing to notice is that, leaving aside the ANNnoTime which we proved to be unable to discriminate the stimuli and the condition, the other two networks without recurrent connections seemed to score better in one condition than in the other. For example, looking at the performances of the standard ANN, while the mean of the correct actions was the highest for the dangerous condition (11,35), in the safe condition it was the lowest (7,97). The inverted pattern can be observed in the ANNColorInvariance, that reached a higher performance in the safe condition. This apparent lack of balance in the results can be explained by the fact that, since the lack of recurrence made the task of associating the action with the outcome slower, the way to obtain an increasing reward at a higher pace was to specialize on the activation of one of the two action neurons, and wait for the weights to balance out thanks to the time tracking of the clock unit. This hypothesis is tested in the multiple comparisons below. A consistent and continuous high performance in both the conditions has been achieved instead by the RNN, with a mean of correct store and discard higher than 10 on the 16 stimuli and scoring around 3 errors. Also, the RNN resulted the only architecture to score 16 on 16 as a maximum of correct actions performed in the two conditions. The balance of performance between the two conditions is not maintained in the case of the RNN without the clock unit, whose performance show instead a specialization pattern comparable to that obtained by the ANNColorInvariant. This can be explained again with the importance of the clock unit as a determinant factor on the adaptation of the architecture, even more than the presence of the recurrence itself. These results are consistent with the fitness plots obtained during the training.

**Table 1:** ANOVA test on the fitness functions.

Dependent Variable: Fitness					
Source	Type III Sum of Squares	df	Mean Square	F	Sig.
Corrected Model	154573,464^a^	4	38643,366	24848,808	,000^a^
Intercept	345922,027	1	345922,027	222437,922	,000^a^
Error	38870,715	24995	1,555		
Total	539366,206	25000			
Corrected Total	193444,179	24999			

The table shows the significance level obtained by the ANOVA test conducted on the fitness scores obtained by the best individual for each condition. a. R Squared =, 799 (Adjusted R Squared =, 799)

^a^Significant values

**Table 2:** Means and std. dev. of the performance divided by stimuli and action.

	ANN	ANNnoTime	RNN	RNNnoTime	ANNColorInv
a) Number of correct actions (store) performed on the safe stimuli					
Mean	*7*,*97*	2,19	10,04	9,58	**11,35**
Std. Dev.	4,532	2,291	4,161	4,451	4,578
Maximum	15	10	16	16	16
b) Number of incorrect actions (discard) performed on the safe stimuli					
Mean	3,72	3,11	2,46	2,60	1,42
Std. Dev.	1,877	2,479	2,197	1,862	1,816
Maximum	9	11	13	9	10
c) Number of correct actions (discard) performed on the dangerous stimuli					
Mean	**11,35**	*3*,*56*	10,30	8,34	8,63
Std. Dev.	4,894	3,258	3,708	3,992	4,339
Maximum	16	13	16	15	15
d) Number of incorrect actions (store) performed on the dangerous stimuli					
Mean	,74	1,46	3,03	3,12	3,80
Std. Dev.	1,247	1,633	1,914	2,090	1,929
Maximum	9	8	9	10	12

The significantly best architecture for each table is highlighted in bold, while the worst is in italic.

What we also aimed to understand is what strategy emerged during the training over generations that allowed the agents to be embedded with an innate circuit that effectively helped discriminate the various conditions in the absence of visual cues, and what patterns of activation of the action units were expressed. Other than that, we examined if there was a difference between the way the robots responded to threatening and safe stimuli. We analyzed the trend of the means of the performances during the life cycle of the best agent of each evolved architecture obtained on the test set (1000 trials) and tried to capture similarities among the expressions of the four principal behaviors: 1) the correct action of storing the safe stimulus (SS); 2) the correct action of discarding the dangerous stimulus (DD); 3) the incorrect action of discarding the safe stimulus (DS); 4) the incorrect action of storing the dangerous stimulus (SD). Considering there is only a maximum of 16 possible actions per behavior in 1000 steps, we condensed the sparse matrix of the recorded answers by dividing the life cycle into 20 intervals of 50 time steps each and averaged the number of responses per behavior in each interval. We conducted a MANOVA test and LSD and Tukey post-hoc analysis on the results.

For brevity, we report only some sections of the LSD post hoc analysis conducted on the standard ANN, but comparable significance levels have been obtained in all the other conditions, except for the ANN deprived of the clock unit. The full post-hoc analysis can be requested separately to the authors. Looking at the significance levels, as showed in Table 3, we observed that there is no significant difference between the means of the SS and SD behaviors in the first interval (steps 0 to 50) while the significance is present between the DD and DS behaviors, even if just slightly below the threshold (p =, 037). This result, consistently with our expectations, displays that the main action performed in all the 1000 trials is the store action, performed on both dangerous and safe stimuli; this strategy allows robots to build up the values of the sensation units and use these input activations as feedback for the next decisions. The store action is the preferred action as it allows the agent to possibly gain both rewards and safety sensation, and shows that the first "assumption" taken by the agents on the environment is that it is safe. These significance levels remain constant until half the life cycle is reached (t = 500), at which point, all the behavior means result significantly different from each other.

**Table 3:** MANOVA post hoc comparisons.

LSD							
Dependent Variable	(I)	(J)	Mean Difference (I-J)	Std. Error	Sig.	95% Confidence Interval	
						Lower Bound	Upper Bound
t (0–50)	SS	SD	,0148	,03409	,665	-,0521	,0816
	DD	DS	-,0713	,03409	,**037**	-,1382	-,0045
t(500–550)	SS	DD	-2,3462	,07566	,**000**	-2,4946	-2,1978
		DS	-2,0865	,07566	,**000**	-2,2349	-1,9381
		SD	,3085	,07566	,**000**	,1601	,4569
	SD	DD	-,3085	,07566	,**000**	-,4569	-,1601
		DS	-2,6547	,07566	,**000**	-2,8031	-2,5063
		DD	-2,3950	,07566	,**000**	-2,5434	-2,2466
T(700–750)	SS	DD	-,2901	,16206	,074	-,6079	,0277

The table shows only the most relevant sections of the MANOVA post-hoc analysis conducted on the performance of the ANN network. SS = means of the store action on the safe stimuli; DD = means of the discard action on the dangerous stimuli; DS = means of the discard action on the safe stimuli; SD = means of the store action on the dangerous stimuli. Significant results are highlighted in bold.

## Discussion

The importance of the presence of a unit signaling time is clearly showed by these results: in fact, both the architectures without the clock scored the lowest and, in particular, the feedforward network deprived of that unit failed to learn at all any strategy to recognize dangerous and safe conditions. In fact, in the first pre-test, the ANNnoTime scored a negative fitness, meaning that it failed to gain any reward in most of the trials and received the abovementioned loss *L = T* instead. The inability for the ANNnoTime to adapt to the situation can be explained by the nature of the feedforward network itself, whose shallow architecture does not allow to keep track of the past unit activations and therefore to associate the state of the sensation units with the outcome of the performed action. While the RNNs can, indeed, learn this association by using the recurrent activations of the action neurons paired with the sensation inputs, the presence of an explicit neuron dedicated to signaling the current time step dramatically speeds up the scaling since its explicit value passed step after step is consistent with that of the reward gain function. The lack of a clock unit can be considered as a model of a lesion in the network in that not just a single ability is impaired, but the overall performance of the robot in his whole lifetime. The interconnections among the neurons and the neural structures, that are the key factors in the variability of the emotional responses in the human brain, are also showing to play an essential role in artificial agents, where the influence of a single input unit can affect the network and therefore the complete generation of a comprehensive response. The emotional response, consistently with our theoretical framework, is also greatly based on environmental variables like spatio-temporal cues and the event timing, so temporal uncertainty can result in a disorganized behavior.

The fitness growth achieved by the best individual of the network tested on both dark and light luminance stimuli, ANNColorInvariance, scored slightly lower than the standard ANN, and this is due to the necessity of the network to process the presence of two different colors. In fact, even if it could be intuitively considered a minor noise, the weight of the changes of the luminance is reflected on all the 49 input neurons of the retina and this slight input variation cannot be cut out completely with one single hidden layer; however, the performance, as will be shown below, is high. This represents a further proof of how a detail in the environment and uncertainty about a situational variable can heavily impact the performance and the way the emotional behavior is displayed.

Considering the general performance of the different architectures, an important element to notice is that, in line with our theoretical framework, while the RNN, equipped with both recurrent connections and the time unit, achieved the best score, the architectures that were lacking one of these two elements exhibited a specialization on a single, mutually exclusive, task. According to the theory of degeneracy and neural reuse, in fact, emotional patterns emerge from the connections between several neural aggregates, each serving a specific function and each necessary but not sufficient to implement the whole affective behavior. A lesion to a part of the circuit, therefore, causes selective deficits, while the ANNnoTime, deprived of all the elements of the networks, could not discriminate the stimuli at all. Other than that, this data confirms that a faster reaction, achieved in the case of the RNN, is crucial to determine the efficiency of the behavior in the environment.

In regard to the analysis of the performances of the best individuals for each condition, while the presence of correlation between the SS and SD action in the early steps can be considered as a strategy brought up to explore the safety of the environment, we can interpret this significant difference between the use of the action units like an attempt of the network to find a new organization, while coping with the internal sensation units and their effect on the hidden connections. So, after the exploration phase is completed, the agent is ready to wait for the right action to be perform when the reward is at its highest. Around time step 700, in fact, that coincides with a nearly total saturation of the previously explained sigmoidal reward function, there is no significant difference between the means of the correct action SS and the correct action DD and between the incorrect actions SD and DS. This last result is particularly explicative in the case of feedforward architectures because it contradicts our hypothesis that the architectures without recurrence could have evolved and significantly specialized on either the discard or store task.

The aim of this experiment has been also to try to shed light on the debate about the necessity of the emotional awareness to display an emotion behavior. With this methodology, we have showed that robots equipped with a unit signaling time were able to efficiently avoid dangerous stimuli. What has emerged in this study is in contrast with the idea of predictive coding, in that robots without prior experience of danger, but evolved throughout generations in risky environments, when equipped with a "complete" structure of the network, were able to correctly exhibit a basic avoidance behavior. Considering that the single robot had not previously gained contact with a harmful stimulus, he could not use information stored ontogenetically nor prior outcomes with similar situations to compute the right pattern to exhibit. The fact that the robots were both "unaware" of the dangerous situation and of its consequences is against a constructionist approach and supports the idea that a cognitive mediation is not required to display a basic emotion response.

Finally, this research gave us insights about the spontaneous emergence of behavioral strategies to maximize the survivability of autonomous agents in dangerous and safe conditions, and proved the importance of the ability of the robots to adapt to different environments using temporal cues coming from their clock unit. We also showed how a phylogenetically selected pattern of action can be expressed and effectively used by evolved agents to guide the growth of ontogenetic fitness in the individual’s lifespan. This leads us to other important questions, such as the possibility to find similarities between the neural patterns of the hidden units of the examined architectures and the structures involved in the genesis of fear and threat detection in humans and animals. Future researches will aim at analyzing the activation patterns of the hidden units, also with the use of lesions, to identify possible modules specialized for the different roles in the genesis of affective behaviors. A current open question is how to map the neural network units to specific brain regions, reproducing not just a basic emotional response but the whole pattern of activation and interconnections able to show a range of meaningful behaviors. Considering that an impairment in negative emotion behavior and recognition is shown after lesions in a wide range of structures, like amygdala [64], insular cortex [65] or cerebellum [66,67], an interesting direction of research could be aimed at understanding the effect of further lesions at the neural level on the behavior shown by robots and on the performance of the different architectures, in order to explore their consistency and similarity to human brain lesions. Furthermore, we wish to investigate the behavior of agents in environments with different percentage of dangerous and safe stimuli to prove the effectiveness of the strategy in uncertain situations and how significant the presence of the time unit proves to be for the achievement of the highest performance on the task.

## Conclusion

We have presented a model of the evolution of fearful behavior and showed the emergence of adaptive strategies during the interaction between the agents and the environment throughout generations using simulated robots inspired on the iCub. We used an experimental setting based on Pavlovian threat conditioning in which the artificial agents were posed in front of a squared board containing safe or dangerous stimuli and were asked to learn to discriminate secure from threatening conditions in the absence of visual cues. Five different neural architectures were evolved with the use of genetic algorithm and the best individuals were tested. We observed, across all architectures and regardless of the presence of recurrent connections, the emergence throughout generations of spontaneous strategies to cope with potentially dangerous environments and the way these behavioral patterns became an innate ability for the evolved individuals to maximize their survivability and reward in their life cycle. We also showed the importance of an internal clock unit as a determinant factor for the fitness and adaptiveness of the individuals.
